# Human Rapid Eye Movement Sleep Shows Local Increases in Low-Frequency Oscillations and Global Decreases in High-Frequency Oscillations Compared to Resting Wakefulness

**DOI:** 10.1523/ENEURO.0293-18.2018

**Published:** 2018-08-29

**Authors:** Benjamin Baird, Anna Castelnovo, Brady A. Riedner, Antoine Lutz, Fabio Ferrarelli, Melanie Boly, Richard J. Davidson, Giulio Tononi

**Affiliations:** 1 Wisconsin Institute for Sleep and Consciousness, Department of Psychiatry, University of Wisconsin–Madison, Madison, WI 53719; 2 Sleep and Epilepsy Center, Neurocenter of Southern Switzerland, Civic Hospital (EOC) of Lugano–Switzerland, Lugano, Switzerland 6903; 3 Lyon Neuroscience Research Center Institut National de la Santé et de la Recherche Médicale Unité 1028, Centre National de la Recherche Scientifique Unité Mixte de Recherche 5292, Lyon 1 University, Lyon, France INSERM U1028, CNRS UMR5292; 4 Center for Healthy Minds, University of Wisconsin–Madison, Madison, WI 53703; 5 Department of Psychiatry, University of Pittsburg, PA 15213; 6 Department of Neurology, University of Wisconsin–Madison, Madison, WI 53705; 7 Waisman Laboratory for Brain Imaging and Behavior, WI 53705

**Keywords:** EEG, REM sleep, sensory disconnection sleep, slow oscillations

## Abstract

It is often assumed that during rapid eye movement (REM) sleep the cerebral cortex homogenously shows electroencephalogram (EEG) activity highly similar to wakefulness. However, to date no studies have compared neural oscillatory activity in human REM sleep to resting wakefulness with high spatial sampling. In the current study, we evaluated high-resolution topographical changes in neural oscillatory power between both early and late naturalistic REM sleep and resting wakefulness in adult humans. All-night recordings with 256-channel high-density EEG (hd-EEG) were collected in healthy volunteers (*N* = 12). Topographic analysis revealed that, compared to wake, both the first and last cycle of REM sleep were associated with increased low-frequency oscillations in local central and occipital regions. In contrast, high-frequency activity in both α and β bands (8–20 Hz) was globally decreased during both early and late REM sleep cycles compared to wakefulness. No significant differences in topographic power in any frequency band were observed between REM sleep cycles occurring early and late in the night. We replicated these findings in an independent dataset (*N* = 33). Together, our findings show that human REM sleep shows consistent topographical changes in oscillatory power across both early and late sleep cycles compared to resting wakefulness.

## Significance Statement

In this work, we present the first high-resolution topographical study of changes in neural oscillatory power between rapid eye movement (REM) sleep and wakefulness in healthy adult humans. Our results show that REM sleep is characterized by globally reduced high-frequency power as well as increased low-frequency oscillations in local regions of the cerebral cortex, including primary sensory, motor and visual cortices. Our findings are consistent with a recent study using laminar recordings rodents, which found that local slow waves occur during REM sleep in middle/superficial layers (layers 3 and 4) of primary visual, sensory and motor areas. Local low-frequency oscillations in primary sensory and motor cortices could be a potential mechanism for disconnection from the external environment during REM sleep.

## Introduction

Since its discovery in the 1950s (Aserinsky and Kleitman, 1953), rapid eye movement (REM) sleep has been a source of intrigue for its “paradoxical” similarity with the waking state. As during non-REM (NREM) sleep, during REM sleep individuals are in a state of quiescence and remain relatively disconnected from the external environment, as demonstrated by strongly attenuated responsiveness to external stimuli. However, in contrast to NREM, in which the brain shows reduced global metabolism (Braun et al., 1997), prominent electroencephalogram (EEG) slow waves, and widespread bistability of cortical membrane potentials (Steriade et al., 2001), during REM sleep the cortex resumes wake-like global activity levels, primarily caused by neuronal activation through brainstem cholinergic projections (Velazquez-Moctezuma et al., 1991). During REM sleep, neurons fire with a similar overall intensity and desynchronized pattern typical of wakefulness, accompanied by similar global levels of blood flow and metabolic activity (Buchsbaum et al., 1989). Visually the scalp EEG of REM sleep, as visualized in sleep polysomnography (PSG), appears similar to wake, exhibiting low-voltage, high-frequency activity.

While it is often stated that neural oscillatory activity during REM sleep is homogenously wake-like, REM sleep notably also includes lower frequency waveforms, such as “sawtooth” waves (2- to 5-Hz waves with a serrated or sawtooth appearance; Sato et al., 1997). Furthermore, recent work has measured brain activity in local regions of cortex during REM sleep using laminar recordings in rodents and found that slow waves, a hallmark of NREM sleep, also occur during REM sleep in localized cortical regions and layers. Specifically, slow waves were observed in primary sensory and motor areas (V1, S1, and M1), but not in secondary visual and motor areas (V2 and M2) or retrosplenial cortex (Funk et al., 2016). Furthermore, this slow wave activity occurred mainly in layer 3 and layer 4, the latter being the main target of relay thalamic inputs to the cortex. As slow waves during NREM sleep are associated with sensory disconnection and higher arousal thresholds (Neckelmann and Ursin, 1993; Ermis et al., 2010), the authors suggested that slow waves in middle/superficial cortical layers of primary cortical regions could partly account for the sensory disconnection that occurs during REM sleep despite the global wake-like activation of the cortex.

The current study had three primary aims. The first aim was to examine narrow-band changes in topographical EEG patterns in naturalistic REM sleep compared to wakefulness in healthy adult humans with high spatial resolution, which, to our knowledge, has not yet been investigated. The second aim was to contrast spatially localized topographical changes in oscillatory power between REM sleep cycles occurring early and late in the night, to test whether neural activation patterns in REM sleep show differences across sleep cycles. Finally, following the results of Funk et al. (2016), the third aim was to examine the spatial distribution of changes in low-frequency oscillations during REM sleep compared to resting wakefulness both on the scalp and on the cortical surface in humans. We examined EEG topographical changes during both the first and last cycle of REM sleep to compare REM sleep cycles with the largest temporal interval (and highest a priori potential to show differences). We used high-density EEG (hd-EEG; 256 channels) coupled with registration to individual cortical anatomy assessed with magnetic resonance imaging (MRI), as this method enables high temporal and spatial resolution and allows for analysis of neural oscillatory activity on the cortical surface.

## Materials and Methods

### Participants

Twelve participants [six females, age = 42 ± 12 (mean ± SD), range 27–59] were randomly selected from a healthy control group participating in a larger sleep research study being conducted at the University of Wisconsin–Madison. Signed informed consent was obtained from all participants before the experiment, and ethical approval for the study was obtained from the University of Wisconsin–Madison Institutional Review Board. All participants were free of significant neurologic conditions as well as sleep-related breathing or movement disorders as verified by PSG. Specifically, all participants had an apnea-hypopnea index (AHI) <10 events per hour and a periodic limb movement arousal index during sleep (PLMSAI) with <15 events per hour of sleep.

### Procedure

Participants completed an all-night hd-EEG recording with simultaneous sleep PSG. Participants arrived at the sleep laboratory between 6 and 8 P.M. hd-EEG and PSG setup lasted ∼2 h. Baseline wake recordings consisted of 6 min of quiet resting wakefulness with eyes closed in a seated upright posture. Participants were instructed to close their eyes, to refrain from moving and to relax while staying awake. Participants went to sleep within 1 h of their most consistently reported bedtime and were allowed to sleep undisturbed until their usual self-reported waking time.

### hd-EEG and PSG sleep recordings

Sleep recordings were made at the Wisconsin Institute for Sleep and Consciousness (University of Wisconsin–Madison) sleep laboratory. hd-EEG recordings were collected using a 256-channel dense array geodesic sensor net (GSN; Electrical Geodesics, Inc.) with a sampling rate of 500 Hz and online referencing to the vertex (CZ). Simultaneous PSG [anterior tibialis, chin electromyogram (EMG), electrocardiogram (EKG), pulse oximetry, pulse oximetry, and respiratory inductance] was recorded in parallel by Respironics Alice5 software (Philips Respironics). Sleep staging was performed offline using standard criteria of the American Academy of Sleep Medicine (AASM; Iber et al., 2007). From the all-night hd-EEG recordings, data segments corresponding to the complete first and last cycle of REM sleep for each participant were selected for further analysis. REM sleep epochs were further divided in tonic and phasic epochs, with phasic REM sleep visually classified by a trained technician as any epochs that contained REMs and/or myoclonic twitches.

### hd-EEG processing

hd-EEG data analysis was conducted with MATLAB (Mathworks Inc.) using the EEGLAB v13 toolbox (Delorme and Makeig, 2004) and custom scripts. EEG data were bandpass filtered from 1 to 30 Hz using a two-way least-squares FIR filter. Data segments and channels containing artifactual activity were visually identified and removed. Consistent with previous studies using 256-channel GSN sensor arrays, electrodes on the face and outer ring of the sensor net were eliminated entirely for all participants due to excessive artifacts, yielding a final scalp montage of 185 channels (Siclari et al., 2014). EEG data were denoised using the Extended Infomax ICA algorithm (Lee et al., 1999). ICA components with specific activity patterns and component maps characteristic of artifactual activity (ocular, cardiographic, and myogenic) were visually identified and removed (Jung et al., 2000). Finally, bad channels were interpolated using spherical splines and EEG signals were referenced to the average of all electrodes.

### hd-EEG spectral analysis

Spectral power density was computed using Welch’s modified periodogram method (*pwelch* function in MATLAB) in 2-s Hamming windows (50% overlap) to decompose EEG time series signals into frequency bands of interest. We analyzed average power spectral density (PSD) in the δ (1–4 Hz), θ (4–7 Hz), α (8–12 Hz), and β (12–20 Hz) frequency bands. Sawtooth waves were visually detected and met the criteria established in Sato et al. (1997): (1) frequency between 2 and 5 Hz, (2) amplitude between 20 and 100 μV, and (3) three or more consecutive waves. We performed a follow-up analysis in which we evaluated whether there was a region-specific correlation between pre-REM δ power and REM δ power to examine whether regions that showed relatively high δ during NREM preceding REM sleep also showed relatively high δ during REM sleep. To conduct this analysis, we first extracted the last 5 min of NREM sleep before REM sleep onset. Then, for each REM and NREM sleep data segment separately, we then computed the *z* score normalized δ power across the scalp and calculated the Pearson correlation coefficient across all participants at each channel.

### MRI acquisition

High-resolution T1-weighted anatomic scans were acquired on a GE 3.0 Tesla MRI scanner before the overnight sleep recording (BRAVO; TR = 6.70 ms; TE = 2.93 ms; TI = 450 ms; flip angle = 12°; FOV = 256 mm; acquisition voxel size = 1 × 1 × 1 mm).

### Source localization

Source modeling was performed using Brainstorm software (Tadel et al., 2011) using cortical reconstructions of individual T1 MRI scans processed using the FreeSurfer pipeline (Fischl et al., 1999, 2004; Han et al., 2006; Jovicich et al., 2006; Fischl, 2012). A symmetric boundary element model (BEM) of the head having three realistic layers (scalp, inner skull, outer skull; Kybic et al., 2005; Gramfort et al., 2010) and a standard coregistered set of electrode positions were used to construct the forward model. The inverse matrix was computed using the minimum norm with sources constrained to be perpendicular to the cortical surface. Spectral power density was computed in source space using Welch’s modified periodogram method in 2-s Hamming windows (50% overlap). From the source PSD, we extracted the magnitude of the complex PSD for further analysis. For group-level whole-brain source analysis, the magnitude of the source PSD was then smoothed at the individual level using a 5-mm full width half max (FWHM) kernel and normalized to the anatomic Montreal Neurologic Institute (MNI) atlas.

### Statistical analysis

Statistical comparisons for sensor and source topographical analysis were made within-subjects and used two-sided paired *t* tests between behavioral states. Group level analyses on average power values were performed separately for each frequency band. At the scalp level, we corrected for multiple comparisons using a nonparametric cluster based permutation test [statistical nonparametric mapping (SNPM); Nichols and Holmes, 2002], with a cluster forming threshold of *t* = 3.12, corresponding to an uncorrected α level of *p* = 0.01. Statistics on cortical sources were computed using the GLM framework implemented in SPM12 (Wellcome Trust Department of Imaging Neuroscience, University College London). Whole-cortex analyses were conducted, correcting for multiple comparisons using topological cluster false-discovery rate (FDR) on the cortical surface. Cluster-size tests were used to test for significant regions using a cluster-forming threshold of *p* = 0.01 and a cluster size threshold of *p* < 0.05 (cluster corrected). Follow-up region of interest (ROI) analysis was performed to examine δ power in primary sensory and motor cortices (S1, M1, and V1). As we had a clear directional hypothesis, one-tailed tests were used for all ROI comparisons. S1, M1, and V1 ROIs were constructed in FreeSurfer using a template derived from histology of postmortem human brains and warped to individual anatomy based on cortical folding patterns (Zilles et al., 2002; Toga et al., 2006; Amunts et al., 2007; Fischl et al., 2008).

### Replication study and comparison to NREM low-frequency oscillations

We repeated our topographical analysis in a separate retrospective group of participants to evaluate the replicability of results obtained from our primary analysis. As the dataset for study 2 was larger and already contained preprocessed data for the whole night, we also used this dataset to perform a follow-up analysis comparing low-frequency oscillatory activity in REM and NREM sleep. Processed EEG/PSG sleep data from thirty-three participants [23 females, age = 49 ± 10 (mean ± SD), range 27–64] were randomly selected from a dataset of a healthy control group participating in a sleep research study being conducted at the University of Wisconsin–Madison. All participants had an AHI with <10 events per hour and a PLMSAI with <15 or more events per hour of sleep. Data acquisition procedures were identical to those described above and included all-night hd-EEG/PSG recordings and baseline waking recordings. EEG data were bandpass filtered from 0.5 to 100 Hz for wake recordings and 1–40 Hz for sleep recordings. We analyzed average PSD in δ (1–4 Hz), θ (4–7 Hz), α (8–12 Hz), and β (12.5–20 Hz) bands, correcting for multiple comparisons using SNPM (cluster forming threshold of *t* = 3.49). EEG processing and spectral analyses were identical to the methods described above. As no anatomic data (MRI) was available for these participants, we did not analyze cortical sources.

## Results

### Global power analysis

**Figure 1.**
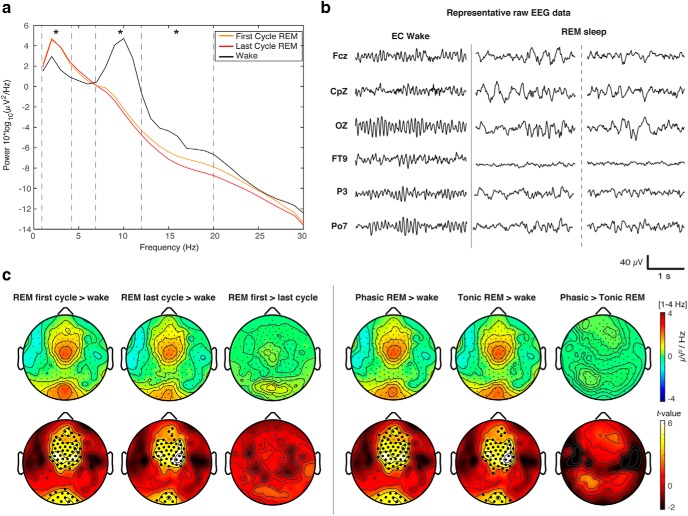
***A***, Global PSD for the first and last cycle of REM sleep and quiet wakefulness. Group average global power spectra (average of all 185 EEG sensors) separated by state (wake (black line), the first cycle of REM sleep (orange line), and the last cycle of REM sleep (red line). Asterisks indicate significant differences between conditions (*p* < 0.05; repeated measures ANOVA) for δ (1–4 Hz), θ (4–7 Hz), α (8–12 Hz), and β (12–20 Hz) frequency bands. ***B***, Representative EEG data across scalp regions for eyes-closed (EC) wakefulness and REM sleep. Central region: Fcz, CpZ; occipital region: OZ; other regions: frontotemporal (FT9), parietal (P3, Po7). ***C***, Topography of REM sleep δ power. Left panel, Topographical differences in δ power [1–4 Hz] in the first and last cycle of REM sleep contrasted with wake. Right panel, Topographical differences in δ power [1–4 Hz] in phasic and tonic REM sleep contrasted with wake. Bottom row, *t* values for all electrodes (two-tailed, paired *t* test); black dots indicate significant differences between states (*p* < 0.05) after correcting for multiple comparisons with SNPM cluster size test.

Sleep architecture variables, including minutes in each sleep stage for individual participants as well as group mean values, are shown in Table 1. Global power (PSD averaged across all EEG sensors) in the δ band was significantly higher in the first cycle of REM sleep (*p* = 0.04) and marginally higher in the last cycle of REM sleep (*p* = 0.08) compared to wakefulness (Table 2; Fig. 1*A*). No significant differences between states in global power in the θ band were observed (*p* ≥ 0.35), while decreased global α and β were observed in both the first and last REM sleep cycle compared to wakefulness (all *p* ≤ 0.01; Table 2; Fig. 1*A*). No significant differences in global power were observed between the first and last cycle of REM sleep (all *p* ≥ 0.09; Table 2; Fig. 1*A*).

**Table 1.** Sleep architecture variables for individual participants and group means

ID	1	2	3	4	5	6	7	8	9	10	11	12	Mean		SD
TSP (min)	391.9	481.9	376.1	486.5	421.9	512.2	428.4	451.6	490.6	470.1	547.4	553.4	467.7	±	56.3
TST (min)	335.2	413.6	352.7	442.2	374.9	427.0	374.5	419.2	364.5	312.0	486.5	500.5	69.5	±	60.6
WASO (min)	57.0	68.5	23.5	44.5	47.0	85.5	54.0	32.5	126.5	158.5	61.0	53.0	85.4	±	40.3
SE	85.5	85.8	93.8	90.9	88.9	83.4	87.4	92.8	74.3	66.4	88.9	90.4	20.5	±	8.3
N1 (min)	15.0	20.0	22.0	9.0	13.5	21.0	17.0	31.5	11.0	17.5	29.5	31.5	19.9	±	7.7
N1 (%)	4.5	4.8	6.2	2.0	3.6	4.9	4.5	7.5	3.0	5.6	6.1	6.3	4.9	±	1.5
N2 (min)	202.0	235.5	172.0	204.0	191.0	185.0	226.0	255.0	112.5	114.0	225.5	198.5	193.4	±	44.0
N2 (%)	60.3	56.9	48.8	46.1	50.9	43.3	60.4	60.8	30.9	36.5	46.4	39.7	48.4	±	9.9
N3 (min)	58.5	85.0	94.5	105.0	71.0	93.0	69.0	32.5	107.0	74.5	73.5	137.5	83.4	±	26.8
N3 (%)	17.5	20.6	26.8	23.7	18.9	21.8	18.4	7.8	29.4	23.9	15.1	27.5	20.9	±	6.0
REM (min)	57.5	71.0	62.0	122.0	97.5	125.5	60.5	98.0	130.5	103.5	155.5	131.0	101.2	±	32.7
REM first cycle (min)	12.5	15.8	34.1	5.9	22.7	11.4	27.7	7.8	11.7	10.6	13.1	22.8	16.3	±	8.6
REM last cycle (min)	10.5	22.6	7.4	7.3	41.0	36.9	33.2	27.8	6.1	38.6	7.9	28.1	22.3	±	13.7
REM (%)	17.2	17.2	17.6	27.6	26.0	29.4	16.2	23.4	35.8	33.2	32.0	26.2	25.1	±	6.9
REML (min)	160.7	113.0	100.4	75.2	13.0	107.4	161.6	71.1	58.1	68.8	101.0	78.1	92.4	±	41.8
AI	9.3	15.7	9.0	7.9	6.2	6.9	9.3	14.3	6.3	4.0	7.9	8.9	8.8	±	3.3

**Table 2.** Global power for the first and last cycle of REM sleep and wakefulness across all frequency bands

	Mean (SD)			*p* value			
Frequency band	REMf	REMl	Wake	REMf > wake	REMl > wake	REMf > REMl	*F* value (*p*)
δ [1–4 Hz]	2.75 (1.02)	2.61 (0.91)	2.11 (0.98)	0.04*	0.08	0.41	4.20 (0.03)*
θ [4–7 Hz]	1.41 (0.55)	1.51 (0.71)	1.61 (1.08)	0.39	0.66	0.35	0.78 (0.39)
α [8–12 Hz]	0.75 (0.07)	0.67 (0.07)	5.12 (1.23)	0.004*	0.004*	0.12	12.91 (0.004)*
β [12–20 Hz]	0.27 (0.19)	0.21 (0.10)	0.58 (0.37)	0.01*	0.003*	0.09	9.23 (0.01)*

* denotes significant differences between conditions.

### hd-EEG topographical analysis

Visual inspection of hd-EEG timeseries revealed that oscillatory activity during REM sleep showed a mixture of both high-frequency, low-amplitude desynchronized patterns as well as low-frequency activity (Fig. 1*B*). Low-frequency activity was predominantly seen in central and occipital scalp regions. Visually, this low-frequency activity appeared as a mixture of different types of waves, including bursts of so-called sawtooth waves as well as slower oscillations, which were at times superimposed with high-frequency activity. Topographic spectral analysis revealed that compared to wakefulness both the first and last cycle of REM sleep was associated with increased δ power in both a central (first cycle: *p* = 0.003, cluster corrected; last cycle: *p* = 0.003, cluster corrected) and occipital (first cycle: *p* = 0.02, cluster corrected; last cycle: *p* = 0.03, cluster corrected) electrode cluster (Fig. 1*C*, left panel). No significant topographical differences in δ power were observed between the first and last cycle of REM sleep (Fig. 1*C*, left panel, right column).

We performed a follow-up analysis comparing REM sleep δ power in the first and last segment of each REM cycle (1st vs last 3rd). No significant clusters were observed (all *p* ≥ 0.19), suggesting that the increased low-frequency power in REM sleep was not attributable to a transitory period of NREM mixture at the beginning of the REM sleep cycle. We next analyzed differences in low-frequency oscillations during phasic and tonic REM sleep (see Materials and Methods). Because no differences were observed between the first and last REM sleep cycle, we collapsed phasic and tonic REM sleep across both the first and last cycle for this analysis. Compared to wakefulness, both phasic and tonic REM sleep had increased δ power in central (phasic: *p* = 0.001, cluster corrected; tonic: *p* = 0.004, cluster corrected) and occipital (phasic: *p* = 0.03, cluster corrected; tonic: *p* = 0.02, cluster corrected) regions (Fig. 1*C*, right panel). No significant differences were observed between phasic and tonic REM sleep in low-frequency oscillations (Fig. 1*C*, right panel).

To analyze differences in REM sleep slow oscillations between REM sleep and wakefulness independently of sawtooth waves, we evaluated differences in low-frequency oscillations between REM sleep and wake after removing REM sleep data segments containing sawtooth wave bursts in either of the central or occipital electrode clusters identified above (see Materials and Methods, hd-EEG spectral analysis). We again observed increased δ power in both central (first cycle: *p* = 0.003, cluster corrected; last cycle: *p* = 0.003, cluster corrected) and occipital (first cycle: *p* = 0.02, cluster corrected; last cycle: *p* = 0.03, cluster corrected) clusters. Additionally, we repeated our spectral analysis for slow oscillations ≤2 Hz and observed that REM sleep showed increased power in the same central and occipital regions for both the first cycle of REM (central cluster: *p* = 0.002, occipital cluster: *p* = 0.01 cluster corrected) and the last cycle of REM (central cluster: *p* = 0.006, occipital cluster: *p* = 0.03, cluster corrected) compared to wakefulness. Finally, we evaluated whether regions that showed relatively high δ power during REM sleep also showed relatively high δ power in the NREM sleep segment immediately preceding the REM cycle (see Materials and Methods, hd-EEG spectral analysis). We found that normalized δ power across the scalp during REM sleep was significantly correlated with normalized δ power during NREM sleep, which peaked over similar central and occipital scalp regions (*p* < 0.05; data not shown).

**Figure 2.**
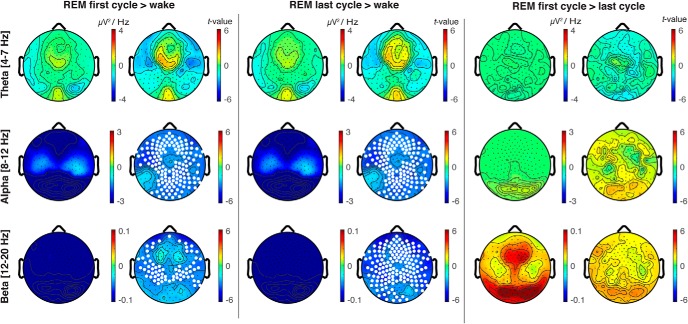
EEG topography of REM sleep contrasted with quiet wakefulness in θ, α and β frequency bands. Topographical differences in oscillatory power between the first cycle of REM sleep contrasted with wake (left panel), the last cycle of REM sleep contrasted with wake (central panel), and the first cycle of REM sleep contrasted with the last cycle of REM sleep (right panel) for θ (4–7 Hz), α (8–12 Hz), and β (12–20 Hz) frequency bands; *t* values are plotted for all electrodes (two-tailed, paired *t* test); white dots indicate significant differences between states (*p* < 0.05) after correcting for multiple comparisons with SNPM.

Decreases in α (first cycle: *p* = 0.003, last cycle: *p* = 0.003, cluster corrected) and β (first cycle: *p* = 0.006, last cycle: *p* = 0.003, cluster corrected) band power were observed in REM sleep compared to wakefulness, but these differences were observed globally and did not localize to specific scalp regions (Fig. 2, left and middle panels). No significant differences were observed between REM sleep and wakefulness in the θ band (Fig. 2). No significant differences in topographic power were observed between the first and last cycle of REM sleep in θ, α, or β frequency bands (Fig. 2, right panel); α and β band power was globally reduced in both phasic and tonic REM sleep compared to wakefulness (all *p* < 0.01, cluster corrected).

### Cortical source analysis

**Figure 3.**
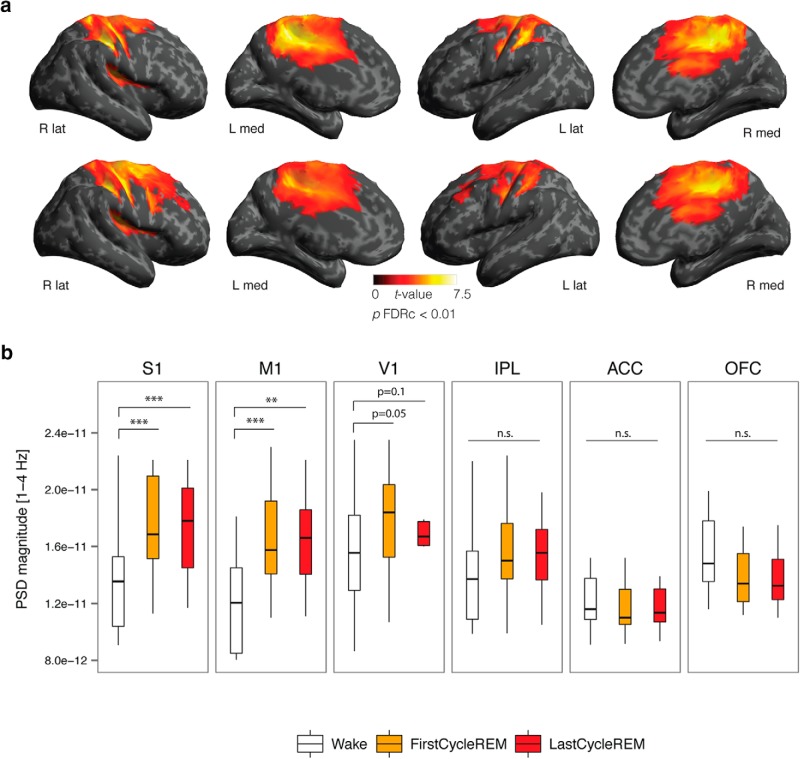
***A***, Source topography of increased δ power [1–4 H] in the first (top row) and last (bottom row) cycle of REM sleep contrasted with wake; *t* values are plotted for all vertices (two-tailed, paired *t* test) exhibiting significant differences between states (*p* < 0.05) after correcting for multiple comparisons using topological FDR cluster correction (height threshold: *p* < 0.01). ***B***, Compared to wake, increased δ power was observed in primary sensory (S1), primary motor (M1), and primary visual (V1) cortices, but was not significantly increased in IPL, ACC, or OFC associative regions. The bottom and top of the boxes show the 25th and 75th percentiles (the lower and upper quartiles), respectively; the inner band shows the median; and the whiskers show the upper and lower quartiles ±1.5× the interquartile range (IQR). Asterisks indicate significant differences between states (*p < 0.05, **p < 0.01, ***p < 0.001; one-tailed paired *t* test).

We followed-up the local topographical increase in δ power in REM sleep by examining differences in low-frequency power at the cortical level. Compared to wakefulness, both the first and last cycle of REM sleep were associated with increased source δ power in a cluster including the right precentral gyrus, postcentral gyrus, posterior cingulate cortex (PCC), and supramarginal gyrus (SMG; *p* < 0.0001, cluster corrected), a cluster including the left precentral gyrus, postcentral gyrus, PCC (*p* < 0.0001, cluster corrected), and the parietal operculum (first cycle: *p* = 0.02, last cycle: *p* = 0.01, cluster corrected; Fig. 3*A*; Table 3). No significant differences in power were observed between the first and last REM sleep cycle at the source level for any frequency band. We also performed a follow-up ROI analysis on primary sensory and motor cortices (S1, M1, V1) in both the first and last REM sleep cycle compared to wakefulness. Compared to wakefulness, increased δ power was observed in S1 (first cycle: *p* = 0.0005; last cycle: *p* = 0.001) and M1 (first cycle: *p =* 0.0005; last cycle: *p* = 0.0005) and marginally increased in V1 (first cycle: *p* = 0.05; last cycle: *p* = 0.10, all one-tailed two-sample *t* tests). In contrast, δ power was not increased in associative cortices of the inferior parietal lobule (IPL; first cycle: *p* = 0.83, last cycle: *p* = 0.85), anterior cingulate cortex (ACC; first cycle: *p* = 0.81, last cycle: *p* = 0.86) or orbitofrontal cortex (OFC; first cycle: *p* = 0.96, last cycle: *p* = 0.97; Fig. 3*B*).

**Table 3.** Source δ power [1–4 Hz] first and last cycle REM contrasted with wakefulness

		Peak MNI			
Region	*p* value cluster	*X*	*Y*	*Z*	*Z* value
First cycle REM > wake					
R postcentral gyrus, R precentral gyrus, R SMG, R PCC	*p* < 0.0001	45	-23	39	4.36
L postcentral gyrus, L precentral gyrus, L PCC	*p* < 0.0001	-9	-25	44	3.77
R parietal operculum	*p* = 0.02	45	-21	18	3.67
Last cycle REM > wake					
R postcentral gyrus, R precentral gyrus, R SMG, R PCC	*p* < 0.0001	38	-20	46	3.79
L postcentral gyrus, L precentral gyrus, L SMG, L PCC, L SMC	*p* < 0.0001	-12	-20	40	3.58
L parietal operculum	*p* = 0.01	50	-21	19	3.53

All clusters significant at *p* < 0.05, FDR cluster corrected (height threshold, *p* < 0.01). SMG = supramarginal gyrus; PCC = posterior cingulate cortex; SMC = supplementary motor cortex.

### Replication study and comparison to NREM

**Figure 4.**
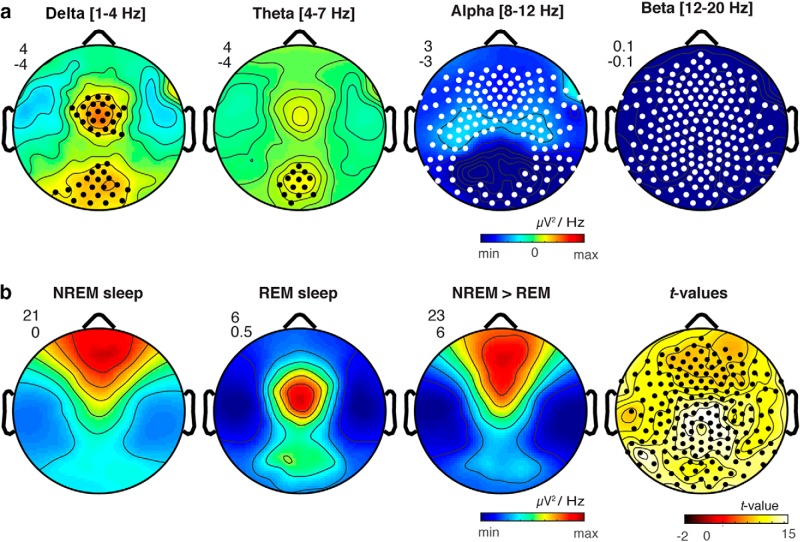
***A***, Replication study. Scalp topography of differences in oscillatory power between REM sleep contrasted with wakefulness in a replication sample (*N* = 33) for δ (1–4 Hz), θ (4–7 Hz), α (8–12 Hz), and β (12–20 Hz) frequency bands. Black dots indicate significantly increased power in REM sleep compared to wakefulness; white dots indicate significant decreases in REM sleep compared to wakefulness (SNPM cluster corrected *p* < 0.05). ***B***, Comparison of REM and NREM δ power. Scalp topography of δ power shown separately for NREM sleep, REM sleep, and NREM > REM sleep as well as *t* values for NREM > REM sleep δ power. The minimum and maximum values for each topographic map are plotted with the corresponding numeric range for the color scale shown in the upper left. Black dots indicate significantly increased power in REM sleep compared to wakefulness (SNPM cluster corrected *p* < 0.05).

We next evaluated whether our topographic results could be replicated in a separate larger group of control participants (*N* = 33) from a different sleep study conducted in our laboratory (see Materials and Methods, Replication study). We again observed that compared to wakefulness REM sleep was associated with increased δ power in both central (*p* = 0.008, cluster corrected) and occipital regions (*p* = 0.02, cluster corrected), as well as increased θ in an occipital cluster (*p* = 0.02, cluster corrected), while α (*p* < 0.0001, cluster corrected) and β (*p* = 0.0003, cluster corrected) were globally decreased (Fig. 4*A*). Together, these data replicate our topographical results with an independent and larger sample.

Finally, we evaluated how δ power in REM sleep compared to δ power in NREM sleep. Replicating previous findings (Riedner et al., 2007), we observed that δ power in NREM sleep was most prominent in prefrontal regions (Fig. 4*B*). This contrasted with the topography of low-frequency oscillations in REM sleep, which, as noted above, showed peaks over middle central and occipital electrodes (Fig. 4*B*). NREM sleep displayed globally increased δ power compared to REM sleep (*p* = 0.02, cluster corrected; Fig. 4*B*). On average REM sleep showed lower δ power as compared to NREM sleep in both the central and occipital electrode clusters, with REM sleep exhibiting 24.4% of NREM δ power in the central cluster and 26.5% of NREM δ power in the occipital cluster.

## Discussion

To the best of our knowledge, the current study is the first to examine topographical EEG patterns in human REM sleep with high spatial resolution and the first to directly contrast spatially localized topographical changes in power in naturalistic REM sleep with wakefulness in healthy adults. Our results show that, compared to wake, both the first and last cycles and both phasic and tonic periods of human REM sleep are characterized by increased low-frequency oscillations in local central and occipital regions. In contrast, high-frequency activity in both α and β bands (8–20 Hz) was globally decreased during both early and late REM sleep cycles compared to wakefulness. No significant differences in local topographic power in any frequency band were observed between REM sleep cycles occurring early and late in the night. Together, these findings show that human REM sleep shows topographical changes in oscillatory power compared to resting wakefulness that are consistent across both early and late REM sleep cycles.

Our results for low-frequency oscillations are consistent with a recent study in rodents, which observed low-frequency activity during REM sleep in primary sensory and motor areas (V1, S1, and M1; Funk et al., 2016). Our results are also consistent with existing human EEG studies, although as noted no previous study has evaluated neural oscillatory activity in human REM sleep with high spatial sampling. In one of the few studies directly comparing EEG power in REM sleep to wakefulness, increased power in the 1- to 6-Hz range in REM sleep was observed, although spatially localized effects were not observed in the 12 channel EEG montage (Corsi-Cabrera et al., 2006). Using slightly improved spatial resolution with a 27-channel montage, Tinguely et al. (2006) observed maximum power in 1- to 6-Hz frequency bands in REM sleep, but local differences in absolute power were not evaluated across states. Another study compared REM sleep with other sleep stages at central electrodes (C3 and C4) and found a higher incidence of δ during REM sleep compared to stage 1 sleep and a lower incidence of δ activity during REM sleep compared to NREM sleep (Armitage, 1995).

Extracellular recordings have revealed that motor cortex is activated during REM sleep (Steriade and Hobson, 1976), and cortical responses to transcranial magnetic stimulation (TMS) administered on the motor cortex are preserved or even increased during REM sleep compared to wakefulness (Hess et al., 1987). Increased regional cerebral blood flow (rCBF) has also been observed in motor cortices during REM sleep in response to learning (Laureys et al., 2001). Similarly, functional neuroimaging (PET and rCBF) studies have also found that visual cortices are active during REM sleep, although the evidence for activation of primary visual cortex is mixed (Maquet et al., 1990; Madsen et al., 1991; Braun et al., 1997). Our results do not contradict these findings. Indeed, it is possible that activation of these areas could coexist with local increases in low-frequency oscillations, particularly if this activity is layer specific, as it is in rodents (Funk et al., 2016).

Intracranial recordings have shown that slow waves during NREM sleep are often spatially restricted, involving only a subset of cortical regions (Murphy et al., 2009; Nir et al., 2011). Furthermore, local slow wave activity has also been observed during waking in both humans and rodents (Vyazovskiy et al., 2011; Bernardi et al., 2015). The current findings are consistent with the local nature of low-frequency oscillations observed in other behavioral states. The mechanisms for the generation of local low-frequency oscillations during REM sleep, however, are not currently understood. Intriguingly, acetylcholine has been found to specifically hyperpolarize layer 4 spiny neurons (Eggermann and Feldmeyer, 2009). If local low-frequency oscillations in primary sensory and primary motor cortices during REM sleep occur mostly in layer 4, as suggested by Funk et al. (2016), it is therefore possible that high levels of acetylcholine during REM sleep could facilitate bistability between ON and OFF periods in these regions. It is also possible that thalamic nuclei could contribute to bistable dynamics in these regions, as thalamic neurons entrain cortical slow waves during NREM sleep (David et al., 2013). While several studies have found that thalamic nuclei show tonic depolarization during REM sleep (Hirsch et al., 1983), low-frequency activity has been unexpectedly observed in intracranial recordings of pulvinar nuclei during REM sleep (Magnin et al., 2004). Overall, more research will be needed to determine the thalamic, cortical and neuromodulatory mechanisms by which local low-frequency oscillations are generated during REM sleep. Additionally, in future research it will be important to evaluate whether this activity is coupled to sleep homeostasis, as is slow wave activity during NREM (Borbély and Achermann, 1999; Riedner et al., 2007). Our finding that the low-frequency activity during NREM sleep preceding REM sleep and during REM sleep are correlated may provide some preliminary evidence for regulation by similar homeostatic mechanisms, although further research is needed to address this important question.

As noted above, a potential functional implication of local low-frequency oscillations in primary sensory and motor cortices is that this activity could partly account for the sensory disconnection that occurs during REM sleep, which has remained a mystery (Nir and Tononi, 2010). At the current time, a mechanistic role for local slow oscillations during REM sleep in sensory disconnection remains speculative. However, there is some evidence for a role of slow oscillations in sensory disconnection during NREM sleep. For instance, slow waves during NREM sleep are associated with higher arousal thresholds (Neckelmann and Ursin, 1993; Ermis et al., 2010). Furthermore, given that bistability between ON and OFF periods impairs cortical information transmission (Massimini et al., 2005; Pigorini et al., 2015), slow wave activity is a plausible mechanism for gating transmission of sensory information to the cortex, particularly when such activity occurs in primary sensory regions. However, an alternative interpretation is that low-frequency oscillations during REM sleep is the result rather than the cause of sensory disconnection, or fulfills another functional role entirely. Sensory disconnection is not always completely blocked during REM sleep and at times auditory and visual stimuli can be perceived, often through incorporation into dream imagery, even while individuals remain asleep (Nir and Tononi, 2010). An intriguing direction for future work would therefore be to directly investigate the role of local low-frequency oscillations in sensory disconnection during REM sleep by testing the relationship between the presence of slow oscillations in primary sensory regions and the probability that sensory stimuli will be incorporated into ongoing oneiric experience.

In the current work, our primary aim was to map narrow-band changes in topographical EEG patterns in naturalistic early and late REM sleep cycles compared to wakefulness. Our research team is currently examining the properties of these waves in detail, which will be important to understanding the nature of these waveforms in greater detail as well as any potential mechanistic role they may fulfill. Specifically, our research team (Bernardi, G., Betta, M., Ricciardi, E., Pietro, P., Tononi, G. & Siclari, F., unpublished observations) is currently characterizing in detail the properties of local low-frequency oscillations in both the central and occipital regions identified here during REM sleep, including the density, amplitude, duration, spatial extent, and negative peaks of individual waves. In line with the remarks above regarding disconnection, in future research it will also be intriguing to investigate whether or how the specific properties of low-frequency oscillations during REM sleep might relate to sensory disconnection.

In summary, our findings show that human REM sleep shows consistent topographical changes in oscillatory power across both early and late sleep cycles compared to wakefulness, consisting of local increases in low-frequency oscillations in central and occipital regions and global decreases in high-frequency oscillations. A speculative hypothesis is that spatially restricted slow oscillations during REM sleep could partially account for the paradoxical nature of REM sleep. Namely, while low-frequency oscillations in primary regions could potentially reduce cortical transmission of bottom-up sensory information, contributing to disconnection (Funk et al., 2016), EEG activation in other cortical regions could facilitate the vivid internal sensory experiences that frequently occur during this state in the form of dreams (Siclari et al., 2017).

## Synthesis

Reviewing Editor: Rae Silver, Columbia University Decisions are customarily a result of the Reviewing Editor and the peer reviewers coming together and discussing their recommendations until a consensus is reached. When revisions are invited, a fact-based synthesis statement explaining their decision and outlining what is needed to prepare a revision will be listed below. Note: If this manuscript was transferred from JNeurosci and a decision was made to accept the manuscript without peer review, a brief statement to this effect will instead be what is listed below.

The authors have expertly addressed each of the concerns raised in the mostly positive previous review of this manuscript. The paper is ready for uploading and publication, though the addition of a visual abstract will be welcome.
